# Assessment of Dapagliflozin Effectiveness as Add-on Therapy for the Treatment of Type 2 Diabetes Mellitus in a Qatari Population

**DOI:** 10.1038/s41598-019-43052-6

**Published:** 2019-05-03

**Authors:** Rana Moustafa Al AdAwi, Zainab Jassim, Dina Elgaily, Hani Abdelaziz, Bhagya Sree, Mohamed Izham Mohamed Ibrahim

**Affiliations:** 1 Pharmacy Department, Hamad General Hospital, Hamad Medical Corporation, Doha, Qatar; 2 Pharmacy Department, Al Wakra Hospital, Hamad Medical Corporation, Doha, Qatar; 3 Women Wellness and Research Center, Hamad Medical Corporation, Doha, Qatar; 4 Social & Administrative Pharmacy, College of Pharmacy Qatar University, PO Box 2713, Doha, Qatar

**Keywords:** Metabolic disorders, Outcomes research

## Abstract

The effectiveness of dapagliflozin in the management of type-2 diabetes mellitus (T2-DM) is an essential issue for establishing a basis for prescribing dapagliflozin. This study aimed to assess the effectiveness of dapagliflozin in combination with other hypoglycemic agents (OHAs) in reducing glycated hemoglobin (HbA1c) and fasting blood glucose (FBG) at 3, 6, 9 and 12 months. This retrospective observational study included all patients who visited the endocrine clinics at Hamad Medical Corporation (HMC) and were treated with dapagliflozin. Demographics and laboratory data were obtained retrospectively from computerized patient medical profiles (eMR-viewer). The main outcome measures were the differences in HbA1c and FBG from baseline at different months. Eighty-one Qatari patients were found to have received dapagliflozin during the study period; 72% of them (n = 58) were males, with a mean age of 57.0 ± 9.0 years and a mean baseline HbA1c of 9.0 ± 1.4%. Administration of dapagliflozin as an add-on therapy was found to decrease HbA1c significantly by 0.8 percentage point after 6 months (P = 0.006) and by 1.5 percentage point after 12 months (P = 0.062). FBG was significantly reduced at 6 months and 9 months (P = 0.001 and P = 0.03, respectively). Dapagliflozin effectively reduced the HbA1c level and FBG when used in combination with other OHAs or insulin within 6 to 12 months.

## Introduction

Dapagliflozin is a sodium-glucose cotransporter 2 (SGLT2) inhibitor, a new class of oral antihyperglycemic drugs with an innovative mechanism of action, and is the second SGLT2 inhibitor to be approved by the Food and Drug Administration of the United States of America (FDA). Managing type 2-diabetes mellitus (T2-DM) with effective and tolerable oral agents will eventually decrease the devastating complications associated with uncontrolled T2-DM and ultimately improve quality of life. In 1990, a novel class of drugs to treat T2-DM with glucose urea was developed but was limited by poor bioavailability due to poor absorption as well as rapid degradation^1–3^. This discovery opened the gate for a promising group of drugs for the treatment of T2-DM, SGLT2 inhibitors, and this group includes canagliflozin, which became the first SGLT2 inhibitor approved by the FDA, followed by dapagliflozin, which was approved by the FDA on January 8, 2014^4,5^.

SGLT2 localizes almost exclusively to the kidney proximal tubules, where it reabsorbs most of the ~180 g of glucose that is filtered through the glomeruli each day^6^. In diabetic patients, the SGLT2 cotransporters are significantly upregulated, increasing glucose reabsorption and leading to glucose conservation and prolonged hyperglycemia. Dapagliflozin is a highly selective and reversible inhibitor of SGLT2 that acts by inhibiting tubular reabsorption of up to half of the glucose filtered by SGLT2 located at segments 1 and 2 in the proximal renal tubule, resulting in a dose-dependent increase in urinary glucose excretion and ultimately, an improvement in glycemic parameters^7–10^. Its C-aryl glucoside-derived chemical structure provides dapagliflozin with a prolonged pharmacokinetic half-life as well as a nearly 3000-fold selectivity for SGLT2 versus SGLT1, making it possible to administer dapagliflozin in an unmodified oral form without affecting SGLT-1-mediated glucose transport in other tissues^11–14^.

This mechanism of action provides us with a valuable clue: dapagliflozin does not act through increasing insulin secretion or decreasing insulin receptor resistance, and thus, commencing therapy with this group of agents neither causes hypoglycemia nor depends on the duration of T2-DM. It can be initiated as monotherapy in newly diagnosed patients or in combination with other oral agents or insulin in patients with long-standing diabetes^15,16^. However, the glucosuria induced by SGLT-2 inhibition might be attributed to hypoglycemia, UTIs, or genital infections^17^.

The efficacy of this novel group of medications requires extensive evaluation in different populations and with different regimens to establish the best practice for managing T2-DM^18,19^. Many studies have evaluated SGLT2 inhibitors and confirmed the efficacy of dapagliflozin. Some studies demonstrated its effectiveness as monotherapy for newly diagnosed patients with T2-DM^13,20–24^. Other groups of studies asserted its effectiveness in combination with other oral hypoglycemic agents^19,25–28^. Moreover, when dapagliflozin was used in conjunction with insulin, studies reported the same level of efficacy with an additional benefit of decreasing insulin demand^28–30^. Of note, dapagliflozin efficacy was well established over a wide range of populations, predominantly in Western populations. Yang *et al*. found that dapagliflozin as an add-on to insulin, with or without oral antidiabetic drugs (OADs) in Asian patients, significantly improved glycemic control^31^. However, to date, there is very few study that has examined the effectiveness of this group of medications in a Middle Eastern population, which has different genetic characteristics^20,32,33^ in addition to unique demographic, culture and lifestyle characteristics^15,34–38^. All these variables may alter the response to SGLT2 inhibitors in general and specifically dapagliflozin.

This study aims to assess the effectiveness of dapagliflozin in the management of T2-DM in combination with other hypoglycemic agents (OHAs) or insulin, in terms of improving HbA1c and fasting blood glucose, among diabetic patients in Qatar. Almost all medicines in Qatar are imported, and the use of the brand originator is high. Thus, establishing evidence of the effectiveness of each medicine in the formulary is important. The results of this study will establish the efficacy of dapagliflozin in the management of T2-DM in Qatar in particular and the Middle East in general.

## Results

### Patient’s profile

All eighty-one patients were Qataris with a mean age of 57 ± 9 years; of the patients, 72% were male. The mean HbA1c and fasting blood glucose at baseline were 9.2 ± 1.4% and 11 ± 4 mmol/L, respectively (Table 1).

**Table 1:** The demographic and clinical characteristics of patients at baseline.

Demographics	n (%)	Mean (SD)
Gender		
Male	58 (72.0)	
Female	23 (28.0)	
Nationality		
Qatari	81 (100)	
non-Qatari	0 (0)	
Age		58 (8.6)
HbA1c		9.2 (1.4)
FBG		11 (4.0)
Co-medications		
Metformin	53 (65.0)	
Pioglitazone	5 (6.2)	
Gliclazide	23 (28.4)	
Glimepiride	15 (18.5)	
Glibenclamide	1 (1.2)	
Repaglinide	1 (1.2)	
Acarbose	1 (1.2)	
Insulin	36 (51.9)	
Vildagliptin	6 (7.4)	
Liraglutide	16 (19.9)	

### Common diabetic treatment combinations

In this study, all patients received dapagliflozin as an add-on therapy in combination with standard diabetic treatment. They were divided into 4 treatment groups based on the most common combinations, and their baseline readings for HbA1c and fasting blood glucose (FBG) were equivalent (p = 0.13 and p = 0.67, respectively) (Table 2).

**Table 2:** The common diabetic treatment combinations.

Treatments groups: N (%)	Medications	A1c (%) at baseline Mean (sd)	FBG (mmol/L) at baseline Mean (sd)
Group 1: 16 (19.8)	Dapagliflozin + Metformin + Sulfonylureas (SUs) +/− DDP4i	9.2	11.8
Group 2: 27 (33.3)	Dapagliflozin + Metformin + DDP4i +/− Insulin	9.9	9.3
Group 3: 10 (12.3)	Dapagliflozin + Insulin + others	10.7	13.6
Group 4: 28 (34.6)	Other combinations Dapagliflozin + (Acarbose, Repaglinide, Liraglutide or Pioglitazone)	9.6	12.6

### HbA1c and FBG levels at the follow-up period

In the follow-up period, repeated measures ANOVA was used to evaluate the difference in HbA1c and FBG following treatment with dapagliflozin. Only values from the 3- and 6-month follow-ups were included, as the number of patients who had data at all time points was very small. Although the overall p-values for the changes in HbA1c and FBG from baseline were significant (p = 0.026 & p = 0.013, respectively) (Table 3), the HbA1c value was significantly lowered from the baseline starting only at the 6-month follow-up (p = 0.045). Similarly, FBG began to decrease significantly at 6 months (p = 0.019).

**Table 3:** HbA1c and FBG levels at baseline and 3 & 6 months posttreatment.

Outcome measures	Baseline (mean/sd)	At 3 months (mean/sd)	At 6 months (mean/sd)	p value
HbA1c*	9.8 ± 0.4%	9.2 ± 0.35%	8.8 ± 0.27%	**0.026**
FBG**	11.4 ± 3.6 mmol/L	9.8 ± 4 mmol/L	8.5 ± 2 mmol/L	**0.013**

*HbA1c baseline to 3 months; p = 0.48. **FBG baseline to 3 months; p = 0.63.

HbA1c baseline to 6 months; p = **0**.**045**. FBG baseline to 6 months; p = **0**.**019**.

HbA1c 3 months to 6 months; p = 0.45. FBG 3 months to 6 months; p = 0.311.

### Changes in HbA1c and FBG from baseline among the different 4 groups

Administration of dapagliflozin was found to reduce HbA1c significantly after 6 months by 1 percentage point (p = 0.045) and showed a decreasing trend after 12 months of 1.5 percentage points (p = 0.062). Moreover, FBG was markedly reduced at 6 months and 9 months (p = 0.001 and p = 0.03, respectively). With further analysis of data from follow-up at 9 and 12 months, the mean HbA1c was reduced by greater values, 1.1 ± 1.4%, and 1.5 ± 2%, respectively; however, these values failed to reach statistical significance (p = 0.88 & p = 0.63, respectively). Likewise, the FBG level was reduced by larger values from 11.0 ± 2.0 mmol/L to 8.0 ± 1.8 mmol/L at 12 months, which could be of clinical importance although not statistically significant (p = 0.7). Notably, the HbA1c (Table 4) and FBG (Table 5) values within the 4 different treatment groups tended to decrease over the 12-month period but failed to reach statistical significance. Even the between-group differences in the HbA1c and FBG readings were statistically nonsignificant.

**Table 4:** The change in HbA1c from baseline among the different treatment groups.

Treatment group	Baseline HbA1c (%)	After 3 months	After 6 months*	After 9 months	After 12 months	P value
Group 1	9.2 (0.64)	8.8 (1.7)	8.4 (1.3)	8.1 (1.2)	8.2 (1.4)	>0.05
Group 2	9.9 (1.6)	8.5 (1.0)	8.8 (1.6)	7.5 (1.7)	7.5 (2.0)	>0.05
Group 3	10.8 (3.2)	9.2 (0.3)	9.7 (0.7)	9.1 (1.3)	8.5 (1.0)	>0.05
Group 4	9.6 (1.3)	9.8 (1.6)	8.5 (0.6)	7.5 (1.0)	7.9 (1.1)	>0.05

Baseline to 6 months between-groups change in HbA1c (P = 0.9)*.

**Table 5:** The change in fasting blood glucose (FBG) from baseline among the different treatment groups.

Treatment group	Baseline FBG (mmol/L)	After 3 months	After 6 months*	After 9 months	After 12 months	P value
Group 1	11.4 (3.2)	12.2 (4.5)	8.8 (13)	8.3 (6)	9.4 (1.8)	>0.05
Group 2	9.7 (2.6)	8.0 (3.1)	8.1 (1.8)	8.6 (0.8)	7.1 (2.1)	>0.05
Group 3	11.9 (4.0)	10.0 (3.7)	10.5 (1.5)	5.9 (0.9)	7.2 (0.07)	>0.05
Group 4	12.2 (4.9)	10.0 (3.2)	7.7 (1.4)	9.7 (1.3)	8.3 (0.9)	>0.05

Baseline to **6** months between-groups change in FBG (P = 0.8)**.

## Discussion

The 2017 American Association of Clinical Endocrinologists (AACE)/American College of Endocrinology (ACE) comprehensive glycemic control algorithm has placed SGLT2 inhibitors before DPP4 inhibitors in the hierarchical order of recommended use as monotherapy as well as add-on therapy^39^. This study was carried out to provide clinicians with research evidence on the use of dapagliflozin and its effectiveness in the management of T2-DM in combination with OHAs or insulin. The findings can be incorporated into their professional judgment and clinical decision-making.

Sufficient evidence demonstrates that many T2-DM patients do not achieve their glycemic goals^40^. T2-DM is a progressive chronic disease, and over time, its treatment requires intensification. Dapagliflozin has a different mechanism of action. According to Fioretto *et al*., dapagliflozin is completely insulin-independent and efficacious as a single therapy or in combination with other agents^40^. In another perspective, Vivian noted that dapagliflozin is recommended as an adjunct to diet and exercise to improve glycemic control^41^. In this retrospective study, information on the diet and physical activities of the patients was not available due to the retrospective nature of the study. The results of this study revealed that dapagliflozin significantly improved the glycemic control of Qatari type 2 diabetic patients when used in combination with standard therapy. The maximum HbA1c reduction of 1.5 percentage points was observed at 12 months, while a significant reduction of 1 percentage point was observed at 6 months from baseline. Fasting blood glucose was reduced started after 6 months of treatment by −2.9 mg/dl (25%). A study by Iijima and coworkers showed that dapagliflozin improved blood glucose levels significantly and worked well in poorly controlled T2-DM patients with glimepiride^42^. In our study, many patients were on glimepiride. Another study in Korea also demonstrated the significant addition of dapagliflozin to an existing drug regimen^43^. The authors also indicated that the use of dapagliflozin as an add-on therapy could be a good alternative for patients who hesitate to use insulin therapy. Notably, the coadministered antidiabetic medications did not influence the reduction in either HbA1c or FBG. Our study was inconsistent with several other studies.

In a randomized study on T2-DM patients who were randomly given dapagliflozin, metformin extended release, or placebo for 12 weeks, List *et al*. found that at the end of the 12 weeks, dapagliflozin induced moderate glucosuria and a significant improvement in the glycemic index. In this study, they established that urinary excretion of 200–300 kcal/day (glucose) is associated with a reduction in HbA1c of 0.5 to 0.9 percentage points and in FBG by 13 to 16 mg/dL from baseline^37^. Additionally, a group of studies evaluated dapagliflozin in combination with metformin slow/extended release^32,37^, glimepiride^32,38^, pioglitazone^13^, sitagliptin^21^, exenatide^44^ and saxagliptin^45^. These studies reported a significant reduction in HbA1c. Moreover, dapagliflozin maintained its efficacy in controlling type 2 diabetic patients on insulin and contributed to reducing their total daily insulin requirement by 50%^29^.

The current study did not include dapagliflozin as a single agent. In a double-blinded, placebo-controlled phase-3 trial, Ferrannini *et al*. administered dapagliflozin as monotherapy to 485 patients inadequately controlled by diet and exercise, with HbA1c between 7.0 and 10%^13^. The patients were randomly assigned to receive placebo or dapagliflozin (2.5, 5, or 10 mg) once daily for 24 weeks. The reduction at the 24th week in HbA1c from the baseline was found to be −0.23 percentage points with the placebo and −0.58, −0.77, and −0.89 percentage points (p = 0.0005, p = 0.0005, and p < 0.0001 respectively) with 2.5, 5 and 10 mg dapagliflozin, respectively. Future studies in Qatar should examine the effectiveness of dapagliflozin as a single therapy.

Although this retrospective study has indicated several significant findings about dapagliflozin, it has some limitations. The patients’ adherence to medication regimen, diet and lifestyle behaviors cannot be measured or followed. The sample size might be too small to confidently generalize the results to all populations who received this drug. Additionally, based on the sample selected, all patients included were Qataris; this fact again affects the generalizability of the results. Despite these limitations, the study has proven that dapagliflozin provides an additional benefit to the reduction in HbA1c and FBG among T2-DM patients in Qatar. Future research should focus on longitudinal follow-up studies with larger sample sizes and inclusion of other segments of the population.

## Conclusion

Dapagliflozin significantly reduced the HbA1c level and FBG of type 2 diabetes patients as add-on therapy, regardless of the type of the coadministered OHA or insulin within a 6-month treatment period.

## Methods

### Study Design

This study is a retrospective before-and-after observational study conducted at Hamad General Hospital (HGH), a tertiary teaching hospital in Qatar. Patients were identified via automated reports generated through the pharmacy dispensing system. All consecutive patients who obtained dapagliflozin from HGH pharmacies during the 2-year time frame from 1 April 2013 until 30 April 2015 were evaluated based on the inclusion and exclusion criteria by reviewing the computerized patient profile system, i.e., eMR-viewer.

### Ethical approval

The study proposal has been reviewed and approved by the Medical Research Center (MRC), an Institutional Review Board at Hamad Medical Corporation (the research ID number is # 15292/15). All methods were performed in accordance with the relevant guidelines and regulations of Hamad Medical Corporation. The ethics committee waived the need to obtain informed consent for this study.

### Study population

All adult diabetic patients who had obtained dapagliflozin from HGH pharmacies during the period of 1 April 2013 to 30 April 2015 were included. Patients were excluded if they were younger than 18 years old, stopped their medication before completing one year, or had inadequate follow-up data.

Eighty-one patients were selected and followed up at 3, 6, 9 and 12 months. Patients were divided into four groups based on the most common antidiabetic medication combinations:

(i) Group 1: Dapagliflozin + Metformin + Sulfonylureas (SUs) +/− DDP4i;
(ii) Group 2: Dapagliflozin + Metformin + DDP4i +/− Insulin;
(iii) Group 3: Dapagliflozin + Insulin + others; and
(iv) Group 4: other combinations: Dapagliflozin + (Acarbose, Repaglinide, Liraglutide or Pioglitazone).

### Study tool

The computerized patient medical profile was accessed to retrieve patient data after receiving ethical approval using a data collection sheet designed prior to the study.

### Data collection procedure

Data regarding prescribed drugs were obtained from the pharmacy dispensing system. Demographic information and laboratory results were obtained from the patient’s computerized profile (eMR-viewer). The period of treatment was determined from the first prescription dispensed from the pharmacy. The data collected included demographics, complete blood count, urea and electrolyte (baseline and follow-up), renal function (baseline and follow-up), duration of treatment, concomitant medications and any discontinuation of treatment.

### Statistical analyses

Descriptive statistics were applied to present the demographics and baseline statistics. For the continuous variables, the mean (SD) or median (IQR) was reported. The categorical data were reported as frequencies and percentages. Data normality was evaluated by the Shapiro–Wilk test. Changes in the mean HbA1c and FBG over the four time points for the normally distributed data were analyzed using repeated measures ANOVA. The paired sample correlation test was carried out to identify points of significant change from baseline. A 95% confidence interval was reported, and a p value ≤ 0.05 was considered indicative of significance unless otherwise noted. Data analysis was performed using SPSS® version 23 (IBM Corp. Released 2013. IBM SPSS Statistics for Windows, Version 22.0. Armonk, NY: IBM Corp) in all analyses.

### Ethical approval

This study was approved by the Institutional Review Board at the Medical Research Center of Hamad Medical Corporation.

### Informed consent

Informed consent was not applicable; data were obtained from the medical records.

## Data Availability

Data are available upon request.
